# Intraoperative suggestions to prevent postoperative delirium in patients undergoing transaortic valvular replacement: a randomized placebo-controlled trial

**DOI:** 10.1007/s40520-023-02476-x

**Published:** 2023-07-01

**Authors:** Christina Kaufmann, Nina Zech, Florian Brandt, Michael Hilker, Kurt Debl, Marcus Creutzenberg, Florian Zeman, Bernhard M. Graf, Barbara Sinner

**Affiliations:** 1 Department of Anesthesiology, University Hospital Regensburg, Regensburg, Germany; 2 Department of Cardiothoracic Surgery, University Hospital Regensburg, Regensburg, Germany; 3 Department of Cardiology, University Hospital Regensburg, Regensburg, Germany; 4 Department of Medical Biostatistics, University Hospital Regensburg, Regensburg, Germany; 5 Department of Anesthesia and Intensive Care, Medical University Innsbruck, Anichstrasse 35, 6020 Innsbruck, Austria

**Keywords:** Intraoperative suggestions, Transcatheter aortic valve replacement, Postoperative delirium

## Abstract

**Background:**

Postoperative delirium (POD) is a serious complication following anaesthesia and surgery and significantly influences postoperative outcome especially in the elderly population. Intraoperative music and positive suggestions influence postoperative outcomes by attenuating analgesic demand and increasing patient satisfaction.

**Aims:**

Here, we examined the effect of intraoperative music and positive suggestions on the development of POD in aged patients undergoing transcatheter aortic valve replacement (TAVR) procedure under general anaesthesia.

**Methods:**

For this randomized placebo-controlled study, eligible patients without cognitive deficit, indicated by a MMSE < 10 points, were anesthetized using remifentanil and sevoflurane. Anaesthetic depth was guide with bispectral index. An audiotape with positive suggestions was applied from a MP3 player via headphones. POD, pain and PONV was assessed. CAM-ICU and Nu-DESC were done twice daily for the first 5 days.

**Results:**

Of 140 patients 118 patients could be analysed (57 male, 80.6 ± 5.1 years). POD was diagnosed in 16 patients (12.7%). POD was significantly more often observed in male (12, 21.1%) than in female (4, 6.6%, p = 0.02) and in patients with a low MMSE (23.6 ± 4.5 vs. 26.8 ± 2.8, p = 0.001). Anaesthetic depth did not influence the incidence of POD. Intraoperative music and suggestions did not affect the rate of POD, pain, analgesic requirement or PONV.

**Discussion:**

In patients undergoing TAVR male sex and low MMSE scoring are associated with an increase in POD.

**Conclusions:**

Intraoperative music and positive suggestions do not influence the incidence of POD in this patient group.

**Study registration:**

DRKS: 00024444, start of registration: 4.02.202, final registration: 17.09.2021

## Introduction

Postoperative delirium (POD) is a serious complication characterized by an acute mental change and fluctuation in attention and cognitive function. POD can result in chronic deterioration of the cognitive functions, postoperative cognitive dysfunction (POCD), and is also associated with increased postoperative morbidity, institutionalization and mortality [1–3]. Several risk factors for the development of POD are discussed among which comorbidity and older age were identified as one of the most important predisposing parameters [3]. Currently, the leading hypothesis suggests that POD is triggered by neurotransmitter imbalance and neuroinflammation [4]. Transcatheter aortic valve replacement (TAVR) is a minimal invasive procedure to replace the aortic valve and is treatment of choice in old patients whose physical status and comorbidity is a relative contraindication for open heart surgery and who are at risk for POD [5]. The systemic inflammation response syndrome occurs more often in patients with open aortic replacement than in TAVR [5].

Even though perception and integration of information during anaesthesia are supposed to be turned off, yet, various observations suggest that auditory information processing is potentially intact [6, 7]. Depending on anaesthetic depth acoustic signals can be perceived. This is utilized when anaesthetic depth is estimated using auditory evoked potentials [8]. Anaesthesia should be deep enough to prevent awareness, active perception or memorizing meaningful events [9, 10]. However, implicit memory, unconscious perception, or memories might also affect the postoperative outcome [11]. There are several studies examining the effect of therapeutic suggestions during general anaesthesia on postoperative outcome [12]. Two meta-analysis of found no positive effect on pain perception and mental distress by positive intraoperative suggestions [12, 13]. However, a positive effect on drug use and recovery could be detected. Nowak et al. examined the effect of intraoperative positive suggestions during general anaesthesia and found a significant reduction in the pain scoring especially during the first two hours following surgery and a reduction in postoperative opioid requirements within 2 and 24 h after surgery compared to control group [14].

Here, we examined the effects of intraoperative positive suggestions applied via headset on the development of POD in aged patients who underwent TAVR under general anaesthesia. Additionally, we evaluated the effect on postoperative pain and nausea and vomiting (PONV).

## Methods

This prospective double-blinded, randomized and placebo-controlled study was approved by the Ethical Committee of the University Hospital Regensburg (No.: 20–2132-101). The study was conducted in accordance with the consolidated standards of reporting trials (CONSORT) guidelines and the principles of Good Clinical Practice, and registered in the German study registration (DRKS, No: DRKS00024444).

### Study design and randomization

Adult patients who underwent elective TAVR procedure under general anaesthesia were considered to be eligible to be included in the study. Patients needed to understand German and hearing disabilities should not exceed the volume adjustment of the MP3 player. Exclusion criteria were neurological disease including severe dementia. Following enrolment of the study, patients underwent neurocognitive assessment using the Minimental State Examination (MMSE). Patients who scored below 10 points were excluded from the study. To ensure correct loudness of the tape the necessary volume of the MP3 player was adjusted using a test tape and documented.

Two MP3 players (Intenso Music Mover™, 8 GB, Intenso GmbH, Vechta, Germany), connected to a headset (Sony MDR-ZX110AP™, Sony, Monato, Japan). One MP3 player contained the study tape and the other with an empty file, were marked with the number 1 or 2. Randomization of the numbers 1 and 2 was performed on a simple randomization base. Each randomization was kept in a sealed envelope. Following inclusion in the study an envelope was assigned to each participant.

Patients in the intervention group listened to a 20-min-long audio file with background music and therapeutic suggestions followed by 10 min of silence, which was played repeatedly until the end of surgery. A different file was played to prepare the patients for emergence from anaesthesia, starting when volatile anaesthesia was stopped. Following hypnotherapeutic principles, the text included direct and indirect positive messages. It addressed topics like competence and care of the surgical and anaesthesiology team, pain regulation, dissociation to a save place, affirmation, anxiety control and confidence. The patients in the control group received an identical looking MP3-player with a blank audio file. For more details please see Nowak et al. [14].

Anaesthesia was induced using Remifentanil (0.1–0.3 μg/kg/min), Oxycodon 50–100 μg/kg, Propofol 1.5–2.5 mg/kg and Rocuronium 0.3–0.5 mg/kg. Anaesthesia was maintained using Remifentanil (0.1–0.3 μg/kg/min) und Sevoflurane (0.6–0.7 MAC). All patients received an arterial line, a central venous line and a pacemaker for rapid pacing. The TAVR procedure was performed in a hybrid operating room and with the use of transoesophageal echocardiography. A balloon aortic valvuloplasty was performed followed by the insertion of a catheter sheath. The bioprosthetic valve, crimped onto a balloon catheter, was advanced across the native aortic valve. During rapid right ventricular pacing, balloon inflation of the crimped heart valve was expanded and was secured to the underlying aortic-valve annulus. Valve position and leak were controlled with TEE. At the end of surgery patients were extubated and transferred to the ICU where they stayed overnight. In general, patients were discharged from ICU the next day.

Following induction of anaesthesia, a Bispectral Index electrode was placed on the forehead and connected to the Bispectral Index monitor (BIS™, Software Version 3.01, Medtronic, Minneapolis, USA) to record anaesthetic depth. BIS was recorded every minute and exported into an excel file. For analysis the mean BIS, BIS episodes below 40 and BIS phases below 40 for more than 5 min were analysed. To determine the relative duration of BIS episodes below 40 the ratio between the duration of recording and the duration of the low BIS episodes was calculated.

A simple randomisation was performed by using the numbers 1 and 2 equally distributed. Each number was stored in a sealed envelope. After successfully finishing the MMSE an envelope was assigned to the patient. Two MP3-players each singed with the number 1 or 2 were used for the study. One with the study and one player the empty control tape. Prior to surgery, the attending anaesthesiologist who was not part of the study team, unsealed the envelope of the respected participant. The respective MP3-player was started and the headset was adjusted to the to the pre-registered loudness and put on the patients´ ears. At the end of surgery, the headset and BIS Monitoring were removed, the patients were extubated and transferred to the intermediate care unit.

POD was detected by physicians who were blinded to the treatment and not involved in the anaesthetic procedure. Patients were tested 2 and 6 h post intervention on the day of surgery, and twice daily on day 1, and 3 in the morning and the evening, respectively and -if the patient developed POD- daily until day 5, using the CAM-ICU (Confusion Assessment Method) and the Nu-DESC (Nurse Delirium Screening Scale). Treatment of the POD was according to the responsible physician. Patients were evaluated for postoperative pain on the day of operation (2 and 6 h post-surgery) and on postoperative day 1 in the morning and the evening using the numerical pain rating scale (NPRS) ranging from 1–10. Analgesic medication was taken from the patients´ charts and was directed by the responsible physician. Patients were also evaluated for PONV, 2, 6 h following extubation and on postoperative day 1. Patient history and pre-existing conditions were taken from the patient charts.

### Statistical analysis

In accordance with previously published data, the sample size was calculated as a 30% rate of patients with POD. Therefore, 58 patients in each group were needed to have 80% power at a two-sided alpha level of 0.05^15^. To allow for potential un-evaluable patients, the number of patients to be enrolled was increased to 140.

The randomization was un-blinded at the end of the patient recruitment. Patient data were stored in a CRF and transferred to a SPSS worksheet (Version 24) for further analysis. BIS data were collected from the BIS Monitor and were stored on an Excel sheet. Continuous variables are reported as mean with standard deviation or interquartile range, when appropriate. Dichotomous variables are reported as numbers with percentages and were compared using the Chi squared test of independence. Non-parametric were analysed using Mann–Whitney U test. Normal distributed categorical variables were analysed with students t-test. Results were statistically significant when p was < 0.05.

## Results

**Fig. 1:**
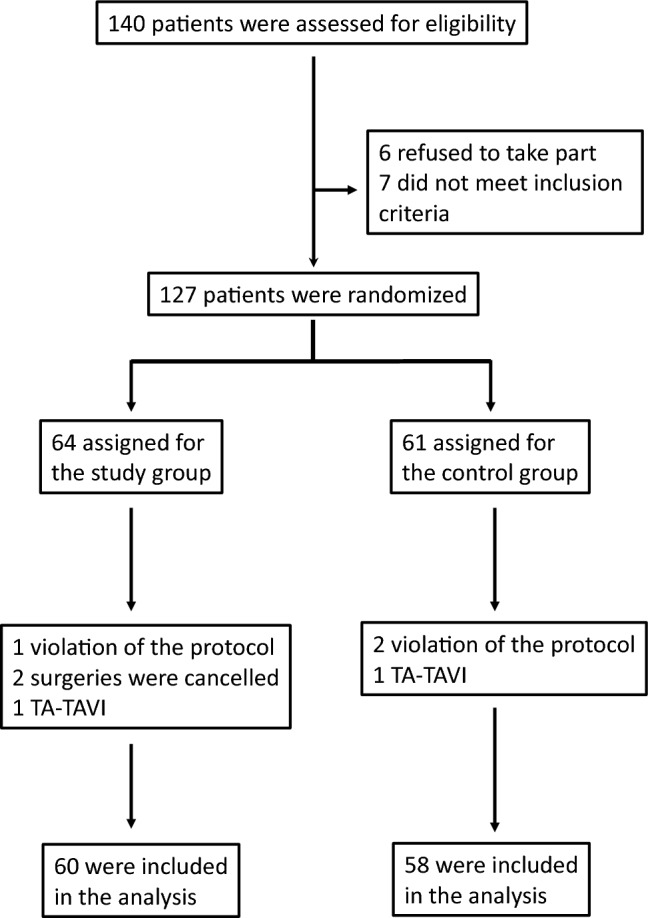
Patient flow-chart

140 patients were evaluated for eligibility (Fig. 1). 126 patients were randomized for either the study (64 patients) or the control group (61 patients). In the study group 4 patients, and the control group 3 patients had to be excluded from the analysis resulting in 118 (57 male, 48.3%) patients (Fig. 1). Mean age was 80.6 ± 5.1 years (range: 65–91) height: 167.6 ± 9.3 and weight: 76.7 ± 14.8). All but two patients were extubated and transferred to the ICU with a mean ICU stay of 2.23 ± 2.0 days (a minimum of 1 day, maximum 9 days, male patients 2.6 ± 2.2 days, female patients 1.9 ± 1.7, p = 0.092). The two patients were extubated on ICU on the day of surgery. Medium length of ICU stay was 2.2 ± 2.0 days (range 1–9 days).

Postoperative delirium was diagnosed using the CAM-ICU in 16 patients (12 men, 12.7%) and using the Nu-DESC in 10 patients (6 men, 7,9%) (Table 1). Patients who were diagnosed with delirium by the Nu-DESC were also diagnosed positive for POD with the CAM-ICU. As the CAM-ICU is more sensitive to diagnose delirium than the Nu-DESC and revealed more delirium in our study than the Nu-DESC, we used the results of the CAM-ICU for further analysis [15].

**Table 1:** Patient characteristics in patients with and without postoperative delirium

	Total	POD	No POD	P
Delirium (CAM-ICU)	118	16 (12.7%)	102 (87.3%)	
Male (n)	57 (48.3%)	12 (21.0%)	45 (79.0%)	0.020 *
Female (n)	61 (51.7%)	4 (6.6%)	57 (93.4%)	
Delirium (NuDESC)		10 (8.5%)	108 (91.5%)	
Male (n)		6	49	
Female (n)		4	57	
Difference between male/female				0.150
Duration of anesthesia (min)	120.3 ± 27.0	119.8 ± 27.1	123.1 ± 27.0	0.352
Duration of surgery (min)	44.0 ± 20.3	43.2 ± 20.0	48.8 ± 22.1	0.481
Length of ICU stay (days)	2.2 ± 2.0	3.5 ± 2.7	2.0 ± 1.8	0.054
Cormorbidities				
Cardiac disease				
Arterial Hypertension (n)	97 (82.2%)	13 (81.5%)	84 (82.4%)	0.581
Arial fibrillation (n)	10 (8.5%)	3 (18.8%)	7 (6.7%)	0.135
Coronary artery disease (n)	84 (71.2%)	10 (62.5%)	74 (72.5%)	0.291
Cong. heart failure (n)	13 (11.0%)	2 (12.5%)	11 (10.8%)	0.555
Pulmonary diseases				
OSAS (n)	14 (11.9%)	1 (6.3%)	15 (14.7%)	0.401
COPD, asthma, emphysema (n)	8 (6.8%)	3 (18.8%)	5 (4.9%)	0.075
Pulmonary hypertension (n)	10 (8.5%)	3 (18.8%)	7 (6.9%)	0.135
Chronic renal failure (n)	67 (56.8%)	11 (68.8%)	56 (54.9%)	0.223
Pre-existing neurologic disease	25 (21.2%)	3 (18.8%)	22 (21.6%)	0.548
Metabolic disease				
Diabetes mellitus (n)	42 (35.6%)	8 (50%)	8 (7.8%)	0.155
Thyroid disease (n)	36 (30.5%)	5 (31.3%)	11 (10.8%)	0.577
MMSE (points)	26,4 ± 3,3	23.6 ± 4.5	26.8 ± 2.8	0.012 *
MMSE 24–10 (mild-moderate dementia) (n)	27 (22.9%)	8 (50%)	19 (18.6%)	0.01*
Male	26.3. ± 3.1	24.0 ± 4.3	26.6 ± 3.1	0.061
Female	26.4 ± 3.4	22.3 ± 5.5	26.5 ± 3.1	0.217

The CAM-ICU was used as reference test for POD. Due to the small number of patients, COPD, Asthma and emphysema as well as all stages of renal failure, respectively, were grouped together. Data are shown as absolute number or mean and SD. Statistical significance with * p < 0.05

The incidence of the postoperative delirium was highest on postoperative day 1 in the morning (9 patients, 7.6%). Men (n = 12, 21.1%) were diagnosed significantly more often (p = 0.02) with a delirium than women (n = 4, 6.6%) and this was still significant after multiple comparisons (p = 0.031). There was no association between the incidence of delirium and the duration of anaesthesia (120.3 ± 27 min, p = 0.352) or the duration of surgery (44.0 ± 21 min, p = 0.481). Besides the aortic stenosis patients were diagnosed with relevant cardiac comorbidities (Table 1). None of the comorbidities were related to the incidence of postoperative delirium. The development of a POD was not associated with an increased length of ICU stay (2.04 ± 1.8 days v. 3.5 ± 2.7 days, p = 0.054). The two patients who could not be extubated immediately after the procedure were not diagnosed with a POD.

The mean scoring in the MMSE was 26.4 ± 3.3 points, with a minimum of 13 points. The attained score did not differ between female and male patients (female: 61, male 57, p = 0.341). Patients who did not develop POD (26.8 ± 2.8) achieved a significant higher scoring in the MMSE (p = 0.0012) than patients who developed a POD (23.6 ± 4.5) at any time point postoperative (Table 1). 22.9% (27 patients, 14 female and 13 male) of the study population presented with a mild to moderate cognitive impairment indicated by a MMSE scoring between 24 and 10. These patients showed a significant increase in POD (p = 0.001) compared to patients with a MMSE of 25 points or higher. Listening to the suggestions and music did not influence the incidence of POD in this subgroup (p = 0.295).

### Anaesthetic depth and burst suppression

BIS values could be recorded continuously in 113 patients (56 male and 57 female). Average BIS was 44.5 ± 6.5. The study protocol demanded that the BIS should be between 60 and 40. However, BIS episodes below 40 for ten seconds or more could be detected in 109 (96.5%) patients (Table 2). In 56 (49.5%) of the patients BIS, was at least once below 40 for more than 5 min. The relative median duration of BIS < 40 was 16.7 (interquartile range: 3.6–40.6). There was no significant correlation between any of the recorded or calculated BIS parameters, respectively, and the development of POD. Four patients (2 female and 2 male) experienced burst suppression during the procedure. Mean duration was (5.5 min ± 4.7 min), (min 40 s, max. 11.5 min). Burst suppression was not associated with delirium (3 patients without vs. 1 patient with POD, p = 0.439) and could not be observed more often in patients with lower MMSE (23.33 ± 3.3 vs. 25.8 ± 1.5, p = 0.58).

**Table 2:** BIS values for all patients and for patients with or without POD

	n	POD	No POD	p
Delirium (CAM-ICU)	113	15 (13.3%)	98 (86.7%)	
Average BIS	44.5 ± 6.5	44.5 ± 5.8	44.5 ± 6.6	0.975
Avarage lowest BIS	26.9 ± 8.7	26.6 ± 5.7	26.9 ± 9.1	0.867
Mean number of BIS phases < 40 for > 5 min	1.0 ± 1.5	1.2 ± 1.7	0.93 ± 1.4	0.556
BIS < 40 (min)	20.9 ± 22.0	22.0 ± 18.8	21.5 ± 20.2	0.942
Median:	14.7	20.9	14	
Relative BIS < 40 (%)	25.4 ± 26.5	24.4 ± 20.2	25.5 ± 27.4	0.995
Median:	16.7	24.3	16.6	
Burst suppression	4	1 (6.7%)	3 (3.1%)	0.439

BIS values could be obtained from 113 patients. The average BIS, the mean of the lowest BIS values as well as the number of BIS phases below 40 during each anesthesia did not differ between patients with and without POD. The relative proportion of BIS phases and the rate of burst suppression was not statistically significant between patients with POD or not, statistical significance with *p < 0.05

Of the 118 patients included in the study group, 60 patients (32 female and 28 male) were exposed to the tape and 58 (29 female and 29 male) were randomized to the placebo group and listened to the empty tape. None of the patients reported of conscious recollection of any event during the TAVR procedure. In each group, 8 patients developed POD. There was no statistically significant difference in POD between the patients who were in the treatment or in the placebo group (p = 0.577).

### Postoperative pain

On the day of the TAVR procedure, 32 patients complaint of pain (NPRS ≥ 3) and average NPRS was 4.4 ± 1.1 (median: 4). On postoperative day one, 7 patients complained of pain (NPRS: 4.6 ± 0.8, median: 4) (Table 3). There was no difference in PRS scoring between patients who were in the treatment and those in the placebo group (day of surgery: p = 0.324, postoperative day 1: p = 0.218). There was also no significant correlation between the treatment group and the control group and consumption of analgesic drugs on both days (Table 3).

**Table 3:**
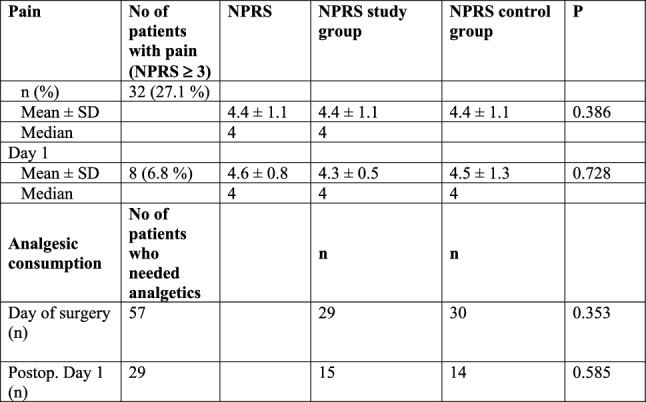
Postoperative pain and analgetic consumption

On day of surgery, a NPRS was recorded in 32 patients. 57 received analgesic drugs following TAVR procedure. In 14 patients analgetic drugs were prescribed without evaluating a NPRS. There was no significant difference in NPRS or pain medication in patients of the study and the control group (p < 0.05 was regarded as statistically significant)

### PONV

Following the procedure, 8 (6.9%) patients complaint of PONV and on day one 1 patient required antiemetic drugs. Listening to the tape did not influence the incidence of PONV (p = 0.517).

## Discussion

This is the first study that examines intraoperative music and positive suggestions on the development of POD. Intraoperative positive suggestions are supposed to have a positive impact on the postoperative process [12–14, 16]. Several studies show that even though postoperative pain is not reduced patients request less pain medication than patients who were not exposed to suggestions and music [12–14]. Here we examined the incidence of POD in aged patients who received positive suggestions and music via an MP3 player during TAVR procedure. POD was more frequently observed in male patients and patients with lower preoperative MMSE. However, intraoperative music and positive suggestions did not influence the incidence in POD.

POD is a serious complication as it impacts postoperative process as well as long-term outcome of the patients resulting in death, dementia, loss of independence and poor cognitive and functional outcome. The incidence in our study was 12.7% and this is in accordance with other investigations reporting an incidence range of 7–26% [5]. There are different screenings tools for delirium. The CAM-ICU has been validated as the most sensitive tool among patients on ICU and is described as more sensitive than other tests [15]. In our study, delirium was also more often detected with the CAM-ICU than with the Nu-DESC. Therefore, we used the CAM-ICU for further analysis.

Several factors were identified who contribute to the development of POD [17]. Male sex is a risk factor for POD [18]. Our results indicate that male patients undergoing TAVR procedure an increased risk for POD. Mauri et al. showed that POD in TAVR patients was a predictor for an increased 2-year mortality [17]. The difference between men and women might in part be due to difference in baseline immune system activity and immune response to stressors [19]. We could not detect a correlation between pre-existing comorbidities and the development of POD, several studies indicate that e. g. chronic heart failure, renal failure or drug abuse are associated with an increase in POD [20].

Another risk factor for POD is preoperative pre-existing neurocognitive decline [1, 2]. We found a strong correlation between the reduction in neurocognitive function indicated by lower MMSE and the development of POD. The low achievements in MMSE can be an indicator for a reduced cognitive reserve to adjust to unfamiliar situations and POD is even regarded as a possible early marker of neurodegenerative or neurocognitive disease [2].

There are conflicting results that depth of anaesthesia and burst suppression is associated with increased rate for delirium [21, 22]. In several studies anaesthetic depth indicated by a BIS below 40 or burst suppression phases were associated with increase in POD [21–23]. We used BIS monitoring to ensure adequate depth of anaesthesia as well as to document low BIS phases and burst suppression. However, we could not show any relationship between low BIS or even burst suppression and the development of POD. Deep anaesthesia in combination with a major surgery and inflammation might promote the development of POD [24]. TAVR is a rather minimal invasive and not painful procedure, however can be associated with a significant systemic inflammation response [25].

There are several studies with mixed results suggesting that therapeutic suggestions during general anaesthesia have a positive impact on postoperative processes [12, 13]. A recently published multi-centre study revealed a significant impact on the use of analgesic drugs but not on pain [14]. Other studies showed a statistically significant correlation between postoperative opioid consumption and audiotape use [13]. Music is more effective when administered before than instead or during the procedure [26]. General anaesthesia alters the conscious reversibly, without turning off the global brain. Anaesthesia therefore might produce different consciousness states ranging from complete absence of any subjective experience to a consciousness experience without perception and memory and episodes of conscious awareness. Some sensory cortex functioning seems to be preserved during general anaesthesia, as the primary auditory cortex remains receptive and reactive to auditory stimuli even during deep sedation [27]. This would allow for implicit awareness, defined as intraoperative unconscious perception without explicit recall. A recent review by Fu et al. assumes that if each consciousness state may potentially be followed by explicit or implicit memories after the procedure. In this respect, anaesthesia can be considered as a “proxy to explore consciousness” [13]. However, in our study in aged patients, music in combination with positive suggestions applied during general anaesthesia did not influence the incidence of POD.

Previous studies report of a positive effect on postoperative analgesic consumption [12–14]. In our study of patients undergoing TAVR procedure suggestions had no effect on postoperative analgesic requirement or pain. This is in contrast to other studies in which Patients undergoing TAVR procedure complaint of modest pain indicated by a low NPRS and the low analgesic demand in our study. In addition, the study was not powered for this and post operative analgesic regime was not standardized but patients received pain medication on demand and the drugs were chosen by the responsible physician. The TAVR procedure is not a painful procedure compared to general surgery and patients mostly complain of pain due to the dressing on the puncture site. In our study, listening to the audiotape had no impact on the development or prevention of PONV. PONV was assessed in several studies. Two studies reported short-lasting PONV relief directly after surgery but not the other day while three studies found PONV to be reduced when patients had been exposed to positive therapeutic suggestions [12].

A limitation of our study is that the contribution of factors other than the therapeutic suggestions and music remain unclear. This includes verbal commands and communication between the persons involved in the TAVR procedure. In addition, the content of the tape is not usefully to prevent POD, e. g. dissociation to a safe place could be rather counterproductive in the context of delirium prevention. Suggestions with more re-orientated content might be more protective. Furthermore, the incidence of POD was rather low. The presumed number of participants was calculated based on the incidence of POD previously described in our TAVR population [28].

In conclusion, in the study in patients undergoing TAVR procedure, POD could be significantly observed more often in male patients and patients with preoperative cognitive decline. The application of intraoperative music and positive suggestions via headset did not reduce the incidence of POD in this patient group.

## Data Availability

Data are currently not available due to ongoing research.

## References

[1] Association AP. American psychiatric association. Diagnostic and statistical manual of mental disorders. American Psychiatric Association. 2013.

[2] Moskowitz EE, Overbey DM, Jones TS (2017). Post-operative delirium is associated with increased 5-year mortality. Am J Surg..

[3] Inouye SK, Marcantonio ER, Kosar CM (2016). The short-term and long-term relationship between delirium and&nbsp;cognitive trajectory in older surgical patients. Alzheimers Dement..

[4] Maldonado JR (2017). Acute brain failure: pathophysiology, diagnosis, management, and sequelae of delirium. Crit Care Clin..

[5] Shi SM, Sung M, Afilalo J (2019). Delirium incidence and functional outcomes after transcatheter and surgical aortic valve replacement. J Am Geriatr Soc..

[6] Cheek DB (1962). The anesthetized patient can hear and can remember. Am J Proctol.

[7] Clark DL, Rosner BS (1973). Neurophysiologic effects of general anesthetics. I. The electroencephalogram and sensory evoked responses in man. Anesthesiology..

[8] Nourski KV, Banks MI, Steinschneider M (2017). Electrocorticographic delineation of human auditory cortical fields based on effects of propofol anesthesia. Neuroimage..

[9] Messina AG, Wang M, Ward MJ (2016). Anaesthetic interventions for prevention of awareness during surgery. Cochrane Database Syst Rev..

[10] Gao WW, He YH, Liu L (2018). BIS monitoring on intraoperative awareness: a meta-analysis. Curr Med Sci..

[11] Levinson BW (1965). States of awareness during general anaesthesia Preliminary communication. Br J Anaesth..

[12] Rosendahl J, Koranyi S, Jacob D (2016). Efficacy of therapeutic suggestions under general anesthesia: a systematic review and meta-analysis of randomized controlled trials. BMC Anesthesiol..

[13] Fu VX, Sleurink KJ, Janssen JC (2021). Perception of auditory stimuli during general anesthesia and its effects on patient outcomes: a systematic review and meta-analysis. Can J Anaesth..

[14] Nowak H, Zech N, Asmussen S et al (2020) Effect of therapeutic suggestions during general anaesthesia on postoperative pain and opioid use: multicentre randomised controlled trial. BMJ 371:m4284. 10.1136/bmj.m4284 10.1136/bmj.m4284 PMC7726311 33303476

[15] Luetz A, Heymann A, Radtke FM (2010). Different assessment tools for intensive care unit delirium: which score to use?. Critical Care Med..

[16] Nilsson U, Rawal N, Uneståhl LE (2001). Improved recovery after music and therapeutic suggestions during general anaesthesia: a double-blind randomised controlled trial. Acta Anaesthesiol Scand..

[17] Mauri V, Reuter K, Körber MI (2021). Incidence, risk factors and impact on long-term outcome of postoperative delirium after transcatheter aortic valve replacement. Front Cardiovasc Med..

[18] Guth AA, Hiotis K, Rockman C (2005). Influence of gender on surgical outcomes: does gender really matter?. J Am Coll Surg..

[19] Ferguson JF, Patel PN, Shah RY (2013). Race and gender variation in response to evoked inflammation. J Transl Med..

[20] Tilley E, Psaltis PJ, Loetscher T (2018). Meta-analysis of prevalence and risk factors for delirium after transcatheter aortic valve implantation. Am J Cardiol..

[21] Brown CH, Edwards C, Lin C (2021). Spinal anesthesia with targeted sedation based on bispectral index values compared with general anesthesia with masked bispectral index values to reduce delirium: the SHARP randomized controlled trial. Anesthesiology..

[22] Soehle M, Dittmann A, Ellerkmann RK (2015). Intraoperative burst suppression is associated with postoperative delirium following cardiac surgery: a prospective, observational study. BMC Anesthesiol..

[23] Pedemonte JC, Plummer GS, Chamadia S (2020). Electroencephalogram burst-suppression during cardiopulmonary bypass in elderly patients mediates postoperative delirium. Anesthesiology..

[24] Taylor J, Parker M, Casey CP (2022). Postoperative delirium and changes in the blood-brain barrier, neuroinflammation, and cerebrospinal fluid lactate: a prospective cohort study. Br J Anaesth.

[25] Zhang K, Troeger W, Kuhn M (2022). Evaluation of systemic inflammation in response to remote ischemic preconditioning in patients undergoing transcatheter aortic valve replacement (TAVR). Rev Cardiovasc Med..

[26] Westmoreland CL, Sebel PS, Winograd E (1993). Indirect memory during anesthesia. The effect of midazolam. Anesthesiology..

[27] Dueck MH, Petzke F, Gerbershagen HJ (2005). Propofol attenuates responses of the auditory cortex to acoustic stimulation in a dose-dependent manner: a FMRI study. Acta Anaesthesiol Scand..

[28] Goldfuss S, Wittmann S, Würschinger F (2019). Anaesthesia-related complications and side-effects in TAVI: a retrospective study in Germany. BMJ Open..

